# The experience of Thai adolescents with depression: A qualitative study

**DOI:** 10.1002/nop2.2161

**Published:** 2024-04-05

**Authors:** Chuntana Reangsing, Khanittha Pitchalard, Katemanee Moonpanane, Pawadee Wimolphan

**Affiliations:** ^1^ School of Nursing Mae Fah Laung University Chiangrai Thailand; ^2^ Nursing Innovation Research and Resource Unit Mae Fah Luaung University Chiangrai Thailand

**Keywords:** adolescent, depression, experience, interpretative phenomenological analysis

## Abstract

**Aim:**

People who have depression experience a maelstrom of emotion as they attempt to understand what is happening to them. While the experience has been quite extensively documented in adults and older individuals, there is a great deal less information available about adolescent depression experiences and reactions. The purpose of this study was to investigate the experiences of Thai‐adolescents suffering from depression.

**Design:**

Interpretative phenomenological analysis.

**Method:**

Fourteen adolescents were recruited from a secondary school in Chiangrai province, Thailand. Semi‐structured interviews were carried out. Interviews were analysed using interpretative phenomenological analysis.

**Results:**

The following four themes were identified: (1) struggling to make sense of their situation, (2) feeling down and withdrawing, (3) contemplating self‐harm and (4) therapy as a last choice. The results point to the continuing significance of promoting psychoeducation for Thai‐adolescents with depression as well as parents, school nurses and health providers while eliminating stigma.

## 1 INTRODUCTION

The fourth‐leading cause of mental illness and impairment among adolescents, depression is a global mental health condition (Bernaras et al., 2019; World Health Organization (WHO), 2021). The global prevalence rate of depression in adolescents varies, for example, 14% in Korea (Yun et al., 2019), and 40% in North India (Singh et al., 2017). Similarly, in the United States, 4.1 million teenagers in the United States between the ages of 12 and 17 are projected to experience at least one major depressive episode in 2020. This number represented 17% of the US population aged 12–17, and about 2.9 million of those had depressive episodes with severe impairment (National Institute of Mental Health (NIMH), 2020). Interestingly, in Thailand, approximately 37.8% of Thai adolescents were categorized at risk for depression and 22% of those had suicide ideation (Patanavanich et al., 2022).

Unfortunately, approximately 60% of adolescents with major depressive disorder have at least one comorbid mental health diagnosis, most commonly anxiety, attention‐deficit hyperactivity disorder (ADHD) (Garcia‐Argibay et al., 2024), conduct disorder, substance use disorders and somatic disorders (Mullen, 2018). Adolescents with chronic medical conditions (e.g., chronic pain, neurological disorders, and autoimmune or inflammatory diseases) also have higher levels of comorbid depression than healthy adolescents (Garcia‐Argibay et al., 2024; Korczak et al., 2023). Among these adolescents, symptoms such as fatigue, decreased concentration, sleep problem and appetite disturbance may overlap with feature of depression, making the diagnosis challenging (Korczak et al., 2023).

Depending on how severe the depression is, different treatments are available. Actually, psychosocial therapies including psychotherapy, interpersonal therapy and supportive therapy can help control mild‐to‐moderate depression, while antidepressants may be necessary for more severe depression (Viswanathan et al., 2020). Only 46% of depressed adolescents obtain treatment, despite the fact that antidepressants and psychotherapy are both powerful remedies, especially when used together. (National Institute of Mental Health (NIMH), 2020). Moreover, approximately 70% of adolescents who have recovered after treatment frequently experience a relapse within 2 years (Mullen, 2018). Furthermore, the statistically significant problem is the length of time it takes for people to receive professional therapy, with many adolescents seeking assistance much later than adults and many years after the commencement of their mental health illness (Otto et al., 2021).

These results could be explained by a variety of factors, such as a fear of stigmatization, cultural differences and challenges in diagnosing adolescent depression. Adolescent depression frequently goes undiagnosed (Stein & Fazel, 2015; Zonca, 2021). This may be due to a difficulty in differentiating warning signs of depression from outdated but persisting views of ‘normal’ teenage behaviours. Additionally, adolescents from different ethnocultural groups would express depression in different ways. For instance, African American adolescents tend to use strong words to express their feelings, such as increased anger, aggression and irritability, while Asian adolescents may represent somatic symptoms to express their depressed feelings (Choi & Gi Park, 2006; Heimann et al., 2018). Thus, it might be difficult for clinicians to detect depression in adolescence. Moreover, there are many factors related to depression in adolescents, especially cultural differences and racial differences. Several researchers found that cultural differences have contributed to distinct aetiology, manifestation, gender difference and treatment of depression in adolescents (He et al., 2021; Kim et al., 2019; McCord et al., 2019; Rose‐Clarke et al., 2021; Xiang et al., 2024). For instance, compared with depression in the West, biological factors, romantic relationships and internet addition lead to a higher prevalence of depression in Chinese adolescent girls than boys (He et al., 2021). Another researchers team found that discrimination, family culture conflict, acculturative and bicultural stress, intragroup rejection, immigration stress and context of reception, which were identified as cultural stressors, were associated with depressive symptoms in Latino adolescents (McCord et al., 2019). Thus, exploring the experiences of depressed adolescents is important and clinicians and health providers should consider the cultural differences. Importantly, diagnostic criteria for depression in the International Classification of Disease (ICD‐10) (World Health Organization (WHO), 2020) and the Diagnostic and Statistical Manual of Mental Disorders (5th ed.; DSM‐5) (American Psychiatric Association (APA), 2013) were primarily formulated based on adult criteria. Adolescents and adults may experience statistically significant depressive episodes with similar fundamental symptoms, but there may be some differences in other ways. For instance, anger and aggression are typical features of depressed adolescents (Genuchi, 2015; Mestre et al., 2017), yet they are not included in diagnostic manuals such as the Diagnostic and Statistical Manual of Mental Disorders (DSM‐V).

In Thailand, in order to establish effective early intervention services for adolescent depression in a Thai‐culture context, understanding the lived experience is crucial because it will help clinicians, health providers and the health policymakers to provide specific strategies to improve depression in Thai adolescents. Interestingly, many researchers examined the prevalence and related factors associated with depression in Thai adolescents (Choychoda et al., 2023; Patanavanich et al., 2022). No prior qualitative study explored the experience of Thai adolescent depression. Therefore, the purpose of this study was to investigate how adolescents who were depressed actually lived. This study is unique as we focused on an Asian population with highly ruralized area.

## 2 METHODS

A hermeneutic or interpretive method called interpretative phenomenological analysis is based primarily on the Heideggerian view of phenomenology, informed data gathering and analysis. The social constructionist viewpoint, which holds that historical, social and environmental element have a major impact on how people experience and perceive their lives, provides the foundation for interpretative phenomenological analysis (Eatough & Smith, 2008, 2017). When a situation is complicated or under investigation, has many facets, or it is difficult to understand, or when a researcher wants to understand how change occurs, this approach might be helpful.

### 2.1 Setting

In this qualitative study, the researchers took samples from a secondary school in Chiangrai province, Thailand. In‐depth interviews were conducted in a private room in a secondary school, Chiangrai province and one individual in‐depth interview was conducted face‐to‐face. This study was conducted from July to September 2022.

### 2.2 Participants

Adolescents with depression were recruited by the school health centre at a secondary school in Chiangrai province, Thailand. The voluntary teacher was informed about this project by the principal investigator; then, the voluntary teacher announced their students for participation. When a participant was interested in this project, a researcher provided information and verified the inclusion criteria. The researcher made several visits to the schools to arrange for parental consents, adolescent assents and depression screening procedures. Adolescents who met inclusion criteria were invited to take part in the in‐depth interview between July and September 2022. Inclusion criteria were (1) youth (age 12–18 years) entering the primary health centre in school with depressive symptoms (depression score >15 via Children's Depression Inventory, CDI screening and they had experienced depression during their adolescence) and (2) having a sufficient command of the Thai language.

Exclusion criteria were (1) a mild cognitive impairment too severe to participate, as assessed by the teacher or interviewer appraisal; the teacher made a clinical judgement about whether it was appropriate to invite adolescents with depression to take part in the study. Possible reasons for not inviting adolescents were, for example, because they were in a state of crisis or because their mental health felt too precarious at this point to ask them to participate in research interviews. Prior treatment was not an exclusion criterion, because researchers were interested in the experience of depressed adolescents in a naturalistic context.

Sixteen adolescents with depression were invited to take part in the study. Of these, 14 agreed to participate. The mean age of those who took part was 15.9 years (SD ± 1.1 years, see Table 1). Ten were female, all of those were single, and 13 resided in the same household as one or both parents; one lived with a grandparent. Five of those were primarily diagnosed with major depressive disorder (*n* = 5).

**TABLE 1:** Socio‐demographic information about depressed adolescents (*n* = 14).

Variables	*n* (%)
Gender	
Female	10 (71%)
Male	4 (29%)
Level of education	
High school year 4	10 (71%)
High school year 5	1 (7%)
High school year 6	3 (22%)
Age	
Mean (SD)	15.9 (1.1)
Range	(15–18 years)

### 2.3 Data collection

Data collection took place in a private room at school. The researchers had an existing relationship with the participants, benefiting the research as there was already a rapport in place, which made participants more comfortable to explore/discuss their sensitive topic. In addition, selecting participants from their vulnerability can assist with engagement. Semi‐structured, in‐depth, audio‐recorded interviews were carried out, each lasting from 45 to 90 min (average 68.5 ± 8 min). The interview was semi‐structured, that is, the researchers had discussed the possible areas of relevance and from this constructed flexible open question. The interviews allowed the researchers to ask a variety of in‐depth questions so that adolescents may express their experiences with depression in their own narrative. There were five main questions in in‐depth interview, namely (1) ‘in your opinion, can you tell me what it is like to be an adolescent with depression?’, (2) ‘can you tell me the ‘bad thing’ if any, about your experience with depression?’, (3) ‘what sign and symptom you have?’, (4) ‘how has the diagnosis of depression impacted on your life?’ and (5) ‘how did you deal with depression?’. We asked follow‐up questions during the interview based on the interview process to obtain more detailed experiences. The guideline questions were reviewed by five experts majoring in adolescent psychology and educational psychology. Examples of interview prompts are listed in Table 2. The researchers summarized the information at the conclusion of each interview to make sure that the participants' viewpoint was accurately expressed and understood, a procedure of verification that enhanced the study's credibility.

**TABLE 2:** Sample of interview prompts relating to the adolescents' experience with depression.

Can you tell me what it is like to be an adolescent with depression? Can you tell me the ‘bad’ thing, if any, about your experience with depression? What signs and symptoms you have? How has the diagnosis of depression impacted on your life? How did you deal with depression?

### 2.4 Ethical considerations

The study was conducted in accordance with the Declaration of Helsinki (as revised in 2013). The study protocol was approved by the Ethical Review Committee for research in human subjects, Chiangrai province, Thailand (No. 35/2565). The data collection procedures were carefully designed to protect confidentiality. All participants were informed verbally and in written form about the study methods and potential risks and benefits of participation. All participants were also informed that their participation was voluntary, and they had the right to refuse or withdraw from participation at any time without impact on them. The adolescents were informed that their interview would be recorded. Once they agreed, the depressed adolescents and their parents were asked to sign a written informed consent. Participants' confidentiality and anonymity were guaranteed throughout the research.

### 2.5 Data analysis

Interpretative phenomenological analysis (IPA) was used to inform the data analysis (Smith et al., 2009). When attempting to understand how people interpret particular experiences, the IPA is a suitable strategy since it enables the individual's lived experiences to be emphasized (Weitkamp et al., 2016). Four members of the research team initially listened to the interview audiotapes to become familiar with the data before coding the interview transcripts. Then, transcripts from each participant were read. The principal investigator identified the content throughout the data collection and analysis processes. The broader codes of struggle, emotion or feelings, therapy, each containing sets of related sub‐codes, were selected based on their prominent appearance in the preliminary review and then were clustered into groups of themes. The consistency of the coded data was checked and rechecked many times during the analysis process by researchers until consensus was reached. Preliminary topics were identified as a result of discussions around emerging ideas. To determine credibility and guarantee saturation, which led to the themes crystallizing, these emerging themes were compared both within and between one another.

## 3 RESULTS

Four overlapping themes were found in the data, representing the adolescents' challenges in understanding and dealing with depression: (1) struggling to make sense of their situation; (2) feeling down and withdrawing; (3) contemplating self‐harm; and (4) Therapy as a last choice.

### 3.1 Struggling to make sense of their situation

In an attempt to comprehend their situation, most adolescents engaged in questioning themselves about why they came with depression, why they were different and why they could not be the same as other adolescents. Below are excerpts that highlight participant's thoughts and feelings about their depression:

Well, I don't know what happened. It happened when I was 12 and everything changed all of a sudden; sometimes I wondered, “Why can't I be like other people?” It' s a negative thought to think about. (Interviewee 2)

Some adolescents are confused about their situation. When the interviewer asked about the cause of depression, most adolescents said they did not know.

I don't know that it had begun when I was 13 years old, I started loving to live alone, I had had not many friends. Every day, I go to school just to study, and then come back to my bedroom, that it. (Interviewee 7)

Recognizing that each person's circumstances were different, particularly in terms of the intensity, duration and effects of their depression, was another facet of their struggle to understand their predicament.

Everything's just harder to get through and then I want to isolate myself from others. I don't like to talk with my parents and sister. They do not understand me at all. (Interviewee 3)

I don't know my situation, it is depression or not, I feel bad most of day and many days in a week. Nobody understand me, I cannot tell everyone. It is so bad. (Interviewee 2)

Some adolescents described how they could not open up to others to share their feelings. These feeling would mostly be suppressed and kept to themselves, while presenting a normal appearance to family members or friends.

It's difficult for me to make a relationship with friends when I am going down. I think I do not need friends; I like to be alone in my space. I sometime do not know how to say/join with friends. I just ask about our homework, that it. (Interviewee 9)

Interestingly, some depressed adolescents did not know how to deal with their thoughts, and feelings. They had no idea for the future. Such as this case:

It is hard…It's gotta be hard in the future. I don't know what to do. (Interviewee 1)

### 3.2 Feeling down and withdrawing

Most adolescents felt that their lives were deteriorating as depression had a greater toll on them. Some adolescents chose to isolate themselves from others by staying in their rooms.

I love to live alone, such as my bedroom, this place is safe for me. You know, when I go out, everybody looks at me and blame!!. (Interviewee 10)

Some adolescents described feeling better when isolating themselves from peers, parents and grandparents because being around others was so emotionally draining.

When I have choices to stay alone or to play/join with friends, I choose living alone. I feel better when I live alone. I don't like to make someone feels bad because of me. (Interviewee 3)

Some students deal with their depressed mood by walking to the hills or going to the public parks to release their emotions or stress. Such as this case:

When I feel bad, I walk to hills. So, my grandpa can't blame me (interviewee 2)

Some adolescents said that they had ceased partaking in things they had previously loved or that they felt unable to fully participate in such activities.

I previously loved to play an online game, it was really fun, but now I do not really enjoy it. It's really good to stay in bed, which really is not like me. My wanting to be in the bed just was not normal. (Interviewee 4)

### 3.3 Contemplating self‐harm

Another consequence of being depressed was that some adolescents thought about harming themselves or committing suicide, especially Thai adolescents diagnosed with major depressive disorder. Four of five Thai adolescents with major depressive disorder had experienced with suicidal attempts (slitting the wrist) as a way of coping with their depression. Such as this case:

When I felt bad, sometimes…. I slit my wrist (interviewee 12)

Some Thai‐depressed adolescents tried to use drug such as alcohol, cannabis and e‐cigarette. This was attributed to a combination of their sufferings due to depression and their perception of how others might react to their situation.

Because of being depressed I have made really stupid choices and done some stuff that's given me a really bad reputation. I started to drink alcohol with strangers. Sometimes, I cracked my skull when I have no idea. (Interviewee 7)

These behaviours of depressed adolescents referred to how they manage or deal with their depression. Also, it indicated that they had ineffective coping strategies, and some clinicians and health providers should be concerned this issue.

### 3.4 Therapy as a last choice

For all adolescents in this study, there was a statistically significant time lag between their first experiencing depression and then seeking professional help. Although the reasons for this varied. In some cases, this was due to a discouraged parent, whereas for some, it was difficult to find a therapist, especially in secondary school with a lack of school nurses. In this study, it appeared crucial for adolescents to make an effort to be independent and demonstrate their independence by overcoming obstacles. They all discussed how they struggled with depression for a protracted period of time prior to seeking help from professionals. It is common belief that therapy is the last choice, only used in the most extreme situations.

“I started to feel bad about myself when I was 13, but it was not going to be worse yet because I had my mom to support me.” “After my mom moved to Singapore, I had nobody to talk to.” “I have no idea where to look for someone to help me.” “I then felt so lonely, and it got worse again after my mom got a new marriage”. “I don't know how to keep better; I did not see the doctor because I am fear to be a psychiatric patient.” (Interviewee 8)

Some students had depression because they experienced chronic stress from bullying. They did not know how to deal with this situation, and their school did not have a school nurse who could help them. They just had to have patience and wait until they moved to another school. Such as this case:

It happened when I was at secondary school (my previous school) because I had been bullied by friends. Every day, they imitated me; it was painful, but there was nothing I could do. I just were patience. Until I moved into this school, I met the teacher even my parent did not accept, and finally I went to consult the doctor, and then my depression was improved. (Interviewee 7)

Some adolescents did not go to see the psychiatrist because her parents did not lose their face, indicating they had a negative perception about depression's treatments. Thus, meeting with a psychiatrist is a last resort for parents to deal with their child's depression. Such as this case:

It happened when I was in grade 8. I started to feel bad when my score was not in the top 3. I have tried to read books a lot, but it doesn't help me. I am so stressed. Dad and mom don't care about my feelings. She needs me to be a doctor…doctor. Nobody cares about me. I really need to see the doctor; I really need help, but dad says no. My dad won't lose face. I felt so lonely. (Interviewee 10)

When I feel down, I really need someone to listen me but you know,…no one helps me. No school nurses here. Nobody…No one know about my bad feelings. I must deal with myself. It is difficult to find someone. I stay in bedroom, cried, and cried…. (Interviewee 7)

Not only did parents not want to lose their faces, but some students also had a negative attitude towards depression treatment. As in this case:

Go to see the doctor is the last choice. You know if I was diagnosed with depression. I cannot be a doctor as I like. Also, it will be too bad if everyone knows I am depressed and take medicine. (Interviewee 2)

## 4 DISCUSSION

Understanding adolescent depression is a complex and challenging task for families, health providers, educators and adolescents themselves. This study aimed to investigate the experience of depression in Thai adolescents. In each of the interview with the adolescents, four interconnected themes have been identified and each will be discussed in turn: ‘struggling to make sense of their situation’; ‘Feeling down and withdrawing’; ‘contemplating self‐harm’; and ‘Therapy as a last choice’.

First, as seen by the theme of engagement to make sense of their circumstances, the adolescents struggled to come to grips with their sadness, which at the same time cast a growing shadow over many parts of their life. The findings suggest that the experience was challenging, demanding, and unpredictable for some adolescents and resulted in emotions of doubt about their present and future. Implicit in this situation was an acknowledgement that something was wrong in their lives. Similarly, a study by Weitkamp et al. (2016) described the experience of depression in adolescents as a ‘black hole’, which illustrates the sense of a loss of control over one's life and the feeling that there is no way out of their situation. Also, some adolescents described themselves with symptoms such as depressed mood, loss of interest, boredom, trouble with making relationships and sleep problems (Weitkamp et al., 2016). For this theme, it is important to clinicians and health professionals to provide information about depression in adolescence including cause, related factors, symptoms and treatment. Also, providing information about how to deal with depression might be effective for reducing the severity level of clinical depression.

Second, many adolescents described their feelings as feeling down and withdrawing. Consistent with a previous study of depression among Arab individuals by Dardas et al. (2019) found that some adolescents reported symptoms of depression as feeling down. Interestingly, our finding is inconsistent with a study by Lesi et al. (2021), which found that about 67.3% of Nigerian adolescents with depression had not recognized social withdrawal from friends as a key symptom of depression. One possible reason might be that there was a cultural difference that resulted in different perceptions of depression. However, in some cultures and Chinese cultures, physical symptoms are more likely to be described (e.g., headaches, ‘heaviness’ and sleep disturbances) for depression (Dere et al., 2013). In this study, as a consequence of struggling to make sense of their depression and feeling down, they protect themselves by withdrawing. Withdrawing helped them to be in a safe zone, increased their confidence and gave them an opportunity to reflect on their situation and try to resolve their problem (Mc Cann et al., 2012). However, withdraw behaviours might not be a good coping strategy for dealing with depression because it might refer to social isolation of depressed adolescents. Importantly, social isolation leads to higher levels of cortisol and worse cognitive functions of depressed adolescents (de Laia Almeidaa et al., 2022). For this theme, it is an issue challenging for clinicians and health professionals to concern during screening and deal with this behaviours of Thai‐depressed adolescents. Providing alternative coping strategies, such as discussing problems with keyperson or family members, like mothers, grandmothers and close friends, might be effective. Family and friends are well placed to provide support which Thai‐depressed adolescents perceive to be positive and which can assist them in obtaining formal mental health treatment (Griffiths et al., 2011).

Third, some adolescents contemplated or attempted self‐harm or suicide, especially Thai adolescents with diagnosed with depression. Our finding consistent with a study by Qaddoura et al. (2022) found that suicidal ideations/attempts were significantly correlated with higher depressive symptoms in Jordanian adolescents. This finding indicated that Thai‐depressed adolescents had maladaptive behaviours in order to cope with their depression. These data suggest that any efforts to address depression among Thai adolescents would benefit from psychoeducation, especially coping strategies. Psychoeducation is the delivery of accurate information to depressed adolescents about depression, its possible causes and symptoms, and its management. Therefore, psychoeducation may help adolescents with depression deal with their depression effectively (Jones et al., 2018).

Fourth, some adolescents claimed that therapy was a last choice, resulting in the well‐known treatment delay for those with depression. This is mirrored in this sample. While some adolescents had a history of seeking therapy, others had endured years of being untreated and kept their burden to themselves. It was feasible to gain some understanding into the causes of the treatment delay because of the qualitative nature of the current study. In some circumstances, the delay was brought on by a lack of knowledge about what is normal, a fear of being stigmatized, or the belief that one should only seek help when things are ‘really bad’. Moreover, if adolescents with depression, the school nurse or the parent failed to convey a sense of urgency, finding a therapist might be even harder.

### 4.1 Strengths and limitations

This study reported here is one of the first to focus on the subjective experience of depression in adolescents and the reasons for treatment delay, particularly in Thai‐speaking country. The adolescents were recruited from secondary school in Thailand and had the opportunity to identify their own experience of depression, which adds to the external validity of the study and warrants consideration of what might be learned from these adolescents. However, it needs to be kept in mind that this study only included those depressed adolescents who agreed to be interviewed and the majority of participants were females. This may partly reflect the greater experience of depression in female depressed adolescents. In addition, all participants were recruited from only rural areas. Therefore, it is possible that we did not see the whole range of depressed individuals in Thailand.

Moreover, a small sample size of depressed adolescents was recruited in this study. Given the schedules of their extremely busy classes, recruitment was difficult. However, depressed adolescents were invited by their teachers and the key themes described emerged in each successive group. Furthermore, some participants in this study were depressed adolescents who were less than 15 years old. Participation in this study required a consent form from their parents. Thus, the recruitment of participants was complex. However, the researcher made several visits to the schools to arrange for parental consent, adolescent consent and depression screening procedures. Thus, all depressed participants were informed that their participation was voluntary, and they had the right to refuse or withdraw from participation at any time without impact on them.

### 4.2 Implications for practice

The findings lead to the question of whether more needs to be done to help depressed adolescents, their parents, school nurses and health providers recognized early warning sign of adolescent depression. Based on the themes in this sample, these warning signs, beyond the established diagnostic criteria, might be a sudden drop in school performance or interpersonal difficulties at school or home. To improve detection, screening tools for depression in adolescents should be developed based on the descriptions of Thai adolescents themselves. Also, when selecting an instrument for depression screening, clinicians should be concerned about the cultural difference. Listening to depressed adolescents would help parents, school nurses and health care providers understand the experience of depression. Also, the school health policymaker would be concerned about cultural differences affecting depression in Thai adolescents and provide psychoeducational interventions focusing on the definition, diagnosis criteria, signs and symptoms, and depression treatments for improving the knowledge, perception and attitude of Thai adolescents with depression and their parents.

## 5 CONCLUSION

To the author's knowledge, this is the first study to explore the experience of Thai‐depressed adolescents. Overall, Thai‐depressed adolescents described findings that it is difficult to understand what was happening to them, feeling alone and withdrawal, self‐harm behaviours and only very lastly considering psychosocial therapies since they believed they had to deal with their depression on their own. These findings suggest many approaches for clinicians and health providers to concern, especially an increasing help‐seeking among Thai‐depressed adolescents including psychoeducation, evidence‐based‐online mental health programme to manage their depression and strategies for reducing stigma associated with depression.

## FUNDING INFORMATION

This work was supported by the Office of the Permanent Secretary, Ministry of Higher Education, Science, Research and Innovation (OPS MHESI), Thailand Science Research and Innovation (TSRI) under Grant No. RGNS 64‐179. Also, this article's processing charges of this work was financially supported by Mae Fah Luang University, Thailand.

## CONFLICT OF INTEREST STATEMENT

No conflict of interest has been declared by the authors.

## ETHICS STATEMENT

The study was conducted in accordance with the Declaration of Helsinki (as revised in 2013). The study protocol was approved by the Ethical Review Committee for research in human subjects, Chiangrai province, Thailand (No. 35/2565).

## Data Availability

Research data are not shared due to the personal nature of the interview data collected and the possibility that participants could be identified.
